# Hereditary gynecological cancer management in women with Lynch syndrome: a survey across Europe

**DOI:** 10.1007/s10689-026-00546-3

**Published:** 2026-03-31

**Authors:** Kevin J. J. Kwinten, Joanne A. de Hullu, ERN GENTURIS Survey Group, Helen Bolton, Monique M. A. Brood-van Zanten, Valentina Chiappa, Stefan Cosyns, Sergi Fernandez-Gonzalez, Angelique Flöter Rådestad, Sophie Frank, Rieke Gellner, Susanna Housmans, Nina Kovacevic, Iveta Mikeltadze, Sebastjan Merlo, Charlotte Møller, Johanna M. A. Pijnenborg, Kresten R. Petersen, Anna Myriam Perrone, Neil A. J. Ryan, Claire Saule, Rawand Salihi, Montserrat Serra Landete, H Lena C van Doorn, Errico Zupi, Lena Steinkasserer, Nicoline Hoogerbrugge, Anne M. van Altena

**Affiliations:** 1 Department of Obstetrics & Gynecology, Radboud University Medical Center, Nijmegen, The Netherlands; 2 Department of Human Genetics, Radboud University Medical Center, Nijmegen, The Netherlands; 3 Department of Gynaecological Oncology, Cambridge University Hospitals NHS Foundation Trust, Cambridge, UK; 4 Department of Gynecology, The Netherlands Cancer Institute, Amsterdam, The Netherlands; 5 Ginecologia Chirurgica, IRCCS Istituto Nazionale Tumori, Milan, Italy; 6 Department of Gynaecologic Oncology, University Hospital UZ Brussel, Brussels, Belgium; 7 Gynaecological Department, Bellvitge University Hospital (IDIBELL), Universitat de Barcelona (UB), Barcelona, Spain; 8 Department of Women’s and Children’s Health, Karolinska Institutet, Stockholm, Sweden; 9 Department of Oncology and Genetics, Institut Curie, Paris, France; 10 Department of Gynecology and Obstetrics, Medical School Hannover, Hannover, Germany; 11 Department Obstetrics & Gynaecology, University Hospitals KU Leuven, Leuven, Belgium; 12 Department of Gynecological Oncology, Institute of Oncology, Ljubljana, Slovenia; 13 Department of Surgical and Gynaecological Oncology, Tartu University Hospital, Surgery Clinic, Tartu, Estonia; 14 Department of Gynecological Oncology, Institute of Oncology, Ljubljana, Slovenia; 15 Department of Obstetrics and Gynecology, Aarhus University Hospital, Aarhus, Denmark; 16 Department of Obstetrics & Gynecology, Radboud University Medical Center, Nijmegen, The Netherlands; 17 Department of Obstetrics and Gynecology, Herlev University Hospital, University of Copenhagen, Herlev, Denmark; 18 Division of Oncologic Gynecology, IRCCS Azienda Ospedaliero-Universitaria di Bologna, Bologna, Italy; 19 College of Medicine and Veterinary Medicine, The University of Edinburgh, Edinburgh, UK; 20 Department of Medical Oncology, Institut Curie, Paris, France; 21 Department of Obstetrics and Gynecology, Ghent University Hospital, Ghent, Belgium; 22 Obstetrics and Gynecology, Hospital Universitari Germans Trias i Pujol, Universitat Autònoma de Barcelona, Badalona, Spain; 23 Department of Gynecologic Oncology, Erasmus MC Cancer Institute, UniversityMedical Centre Rotterdam, Rotterdam, The Netherlands; 24 Department of Molecular and Developmental Medicine, Obstetrics and GynecologicalClinic, University of Siena, 53100 Siena, Italy; 25 Department of Gynaecology and Obstetrics, Hannover Medical School, Hannover, Germany

**Keywords:** Lynch syndrome, Gynecological cancer, Guidelines, Hereditary cancer, Cancer genetics, Multidisciplinary care

## Abstract

We aim to compare clinical practices in the gynecological management of women with Lynch syndrome across Europe and assess the need for multidisciplinary gynecological care for this high-risk population. European gynecologists involved in the clinical care of women with Lynch syndrome were invited to complete a cross-sectional survey regarding their clinical practices and organization of care. All participating gynecologists were based in hospitals that are members of the European Reference Network on Genetic Tumour Risk Syndromes (ERN GENTURIS). A total of 33 gynecologists (57%) from 25 unique hospitals across 12 countries participated in the survey. National guidelines on the gynecological management of women with Lynch syndrome were absent in a significant number of European countries. Local multidisciplinary team meetings were available in 61% of hospitals. Considerable heterogeneity was reported in the organization of care, as well as in surveillance, prevention, and treatment strategies. Standardized European guidelines for the gynecological care of women with Lynch syndrome are warranted to promote uniformity. Gynecologists specialized in hereditary cancer should take the lead in developing and implementing these guidelines. Hospitals managing women with Lynch syndrome should have the resources to organize multidisciplinary team meetings, which are crucial for the effective implementation of these guidelines and the improvement of personalized care.

**Supplementary Information:**

The online version contains supplementary material available at 10.1007/s10689-026-00546-3.

## Introduction 

Lynch syndrome is a hereditary cancer syndrome caused by a germline pathogenic variant (gPV) affecting one of the four DNA MisMatch Repair (MMR) genes *MLH1*, *MSH2*, *MSH6*, and *PMS2* [1]. Women with Lynch syndrome have a high hereditary risk of developing colorectal cancer, endometrial cancer, and less frequently, other Lynch syndrome-associated cancers such as ovarian, gastric, duodenal, pancreatic and urinary tract cancer [2]. The lifetime risks to develop endometrial and ovarian cancer vary by the affected MMR-gene, ranging from 21-46% for endometrial cancer and 2-10% for ovarian cancer [3].

Colorectal surveillance with colonoscopy is recommended to early detect precursor lesions in the colorectum [4]. European gastrointestinal guidelines recommend gene-specific surveillance intervals and initiation ages, typically starting at age 25 or 35, with intervals of 2 to 5 years depending on the gene involved [5–7]. The 2025 revised multidisciplinary Dutch guideline on colorectal cancer surveillance in Lynch syndrome recommends biennial colonoscopy starting at age 25 for carriers of a gPV in *MLH1* and *MSH2*, at age 30 for *MSH6*, and at age 35 for *PMS2* [8].

Endometrial surveillance in Lynch syndrome, using transvaginal ultrasound and endometrial biopsy, may be offered to detect premalignancies or early-stage endometrial cancer; however, no survival benefit has yet been demonstrated [9, 10]. Endometrial cancer generally has a favorable prognosis because the majority is diagnosed at an early stage due to the presence of early symptoms. Irregular uterine bleeding in premenopausal women and postmenopausal blood loss are the most prominent symptoms [11]. Women with Lynch syndrome are therefore advised to consult a gynecologist if they experience such symptoms [9].

In the general population, as well as in high-risk populations, ovarian surveillance with ultrasound and/or the biomarker CA125 has proven to be ineffective for early detection of ovarian cancer [12, 13]. Yet, recommendations for gynecological surveillance in Lynch syndrome still vary among countries [9, 14].

Risk-reducing total hysterectomy with bilateral salpingo-oophorectomy (BSO) may be considered for women with a completed or no child wish to reduce the risk of endometrial cancer and ovarian cancer [9, 15]. However, a hysterectomy is major surgery and therefore associated with potential peri- and postoperative complications [16]. BSO in premenopausal women results in premature surgical menopause, which can lead to severe menopausal symptoms, psychosexual problems, and an increased risk of cardiovascular disease, osteoporosis, and cognitive disorders [17, 18]. The favorable prognosis of endometrial cancer, the lack of high-quality data about gynecological surveillance, and the high impact of risk-reducing surgery, add to the variation in recommendations on gynecological cancer care in women with Lynch syndrome.

In this study, we describe the current clinical gynecological management of women with Lynch syndrome across Europe. Our aim is to facilitate the comparison of clinical practices and assess the need for multidisciplinary gynecological care for women with Lynch syndrome.

## Material and methods

### Study design

This study was designed as a cross-sectional survey targeting European gynecologists involved in the clinical care of women with Lynch syndrome. Participants were invited to complete a structured questionnaire regarding their clinical practices and organization of care.

### Survey development

The survey was developed by a gynecologic oncology researcher (KK), two gynecologic oncologists (JH and AA), and a hereditary cancer specialist (NH). It was independently reviewed by an external gynecologic oncologist to ensure clarity and relevance. The questionnaire covered seven key domains: (1) general information, (2) organization of care, (3) guidelines, (4) in clinical practice: surveillance strategies, (5) in clinical practice: management of cancer prevention, (6) in clinical practice: treatment of (pre)malignancies, and (7) patient participation. The majority of questions were quantitative, with optional open-text fields provided for comments in each domain. The full survey instrument is available in Supplementary File 1.

### Study setting and data collection

Gynecologists working in institutes that are member of European Reference Network on Genetic Tumour Risk Syndromes (ERN GENTURIS) were invited to participate. ERN GENTURIS is a network of expert centers for hereditary cancer across European member states, aiming to improve identification of people with a genetic tumor syndrome, reduce variation in clinical practice in Europe, and develop evidenced based clinical guidelines [19]. The network consists of healthcare providers working in 51 expertise centers across 22 European Union member states and Norway, and supporting partners working in 3 expertise centers in the United Kingdom.

The designated representative of each ERN GENTURIS center was asked to provide contact details of two gynecologists involved in the care of women with Lynch syndrome. These gynecologists were subsequently invited to participate in the survey. The survey was distributed via Castor Electronic Data Capture (EDC) between May and July 2024.

## Results

1. General information

A total of 33 (57%) of 58 gynecologists completed the survey. The majority (67%) of them was female with a median of fifteen years of experience as a gynecologist. Nineteen (57%) were gynecologic oncologist with a completed fellowship, seven (21%) gynecologists with an interest in oncology, and seven (21%) were general gynecologists. Most of them (94%) were working in a university hospital, and 73% identified their hospital as a specialized hereditary cancer clinic. Participants worked in 25 unique hospitals in Belgium (4), Denmark (3), Estonia (2), France (2), Germany (3), Italy (4), the Netherlands (7), Portugal (1), Slovenia (2), Spain (2), Sweden (1), and the United Kingdom (2).

2. Organization of care

**Fig. 1:**
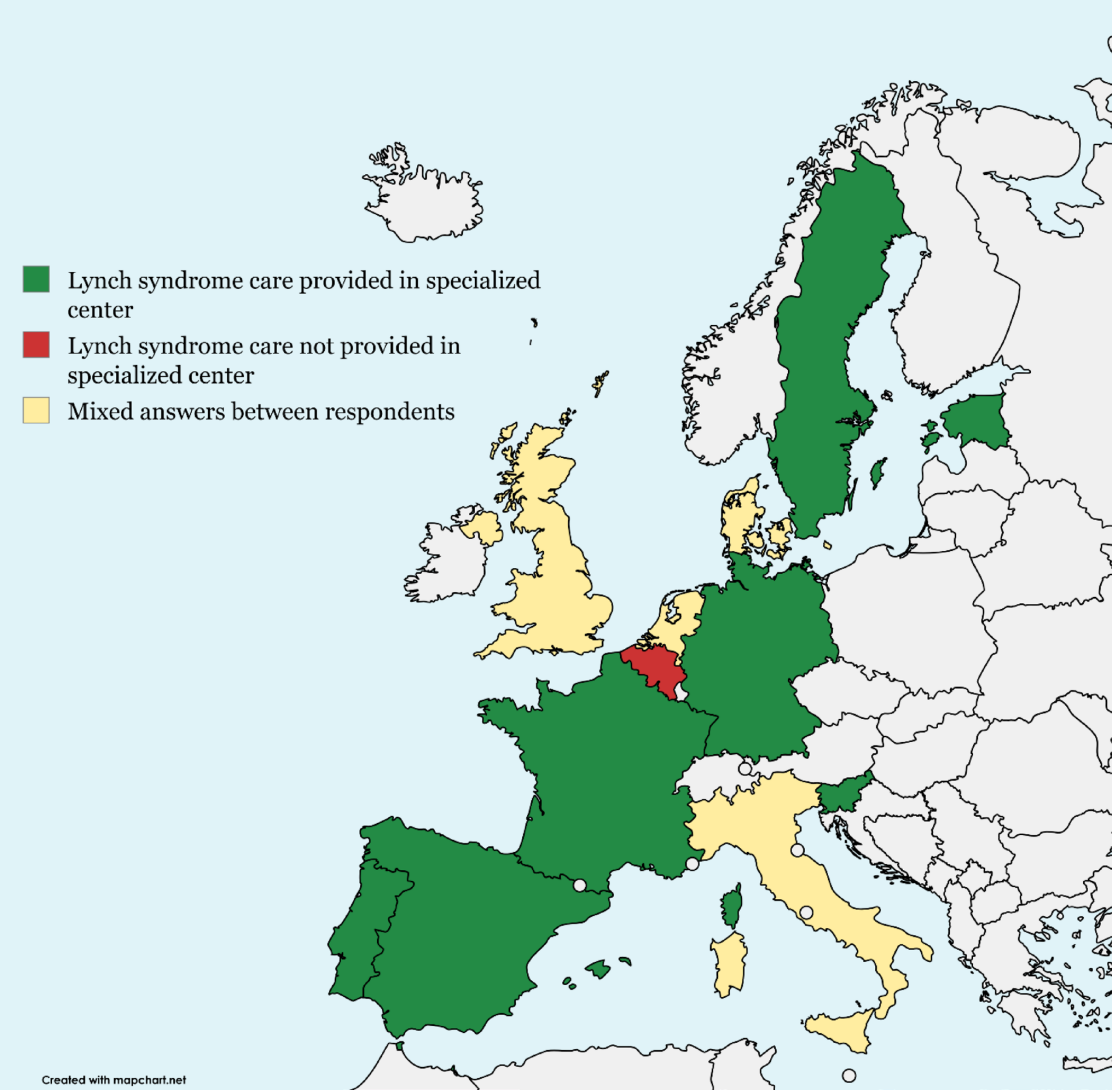
Type of clinic where gynecological care in women with Lynch syndrome is generally provided across Europe.

The type of clinic providing care for women with Lynch syndrome varies across and within European countries (Figure 1). In seven countries, all gynecologists reported that care is provided in specialized Lynch syndrome centers. Gynecologists from four countries gave mixed responses to whether care for women with Lynch syndrome is organized in specialized centers within their country.

The organization of local multidisciplinary team meetings (MDTs) for (complex) patients with a hereditary tumor syndrome is not standardized in every hospital or expert center across Europe. Among the 33 respondents, twenty (61%) reported MDTs in their clinic. In half of these clinics, only complex patients are discussed. The most frequently represented specialties in MDTS include gynecology (85%), clinical genetics (80%), gastroenterology (60%), pathology (60%), medical oncology (55%), and colorectal surgery (5%).

3. Guidelines

Gynecologists are aware of the existence of a national gynecological guideline related to Lynch syndrome in nine of twelve countries (Table 1). Nonetheless, discrepancies exist among gynecologic specialists within countries regarding awareness of the existence of these guidelines. In five of the twelve countries, respondents provided conflicting information about whether a national guideline is available.

**Table 1:** Overview of Lynch syndrome-related gynecological national guidelines

	Existence of national guideline	Endometrial surveillance	Ovarian surveillance	Recommendation of multidisciplinary team meetings
BE (4)	Mixed (2Y, 1N, 1UNK)	TVU (2) + EMB (1), EMB if aberrancy (1)	TVU (2) + CA125 (1)	No (2)
DE (3)	Yes (3)	TVU (3) + EMB if aberrancy (2)	TVU (3)	Mixed (2N, 1UNK)
ES (2)	No (2)			
FR (2)	Yes (2)	TVU + EMB (2)	TVU (2)	Yes (2)
GE (3)	Mixed (2Y, 1N)	TVU + EMB (2)	TVU (2)	Yes (2)
IT (4)	Mixed (2Y, 2N)	TVU (2) + EMB (1), EMB if aberrancy (1)	TVU (2)	Mixed (1N, 1UNK)
NL (7)	Mixed (6Y, 1N)	TVU (5) + EMB (5), UNK (1)	TVU (1), no (4)	Mixed (3N, 2UNK)
PO (1)	No			
SL (2)	No (2)			
SP (2)	Mixed (1Y, 1N)	TVU + EMB (1)	TVU (1)	Yes (1)
SW (1)	Yes	TVU + EMB if aberrancy	TVU	Yes
UK (2)	Yes (2)	TVU + EMB and hysteroscopy (1), no (1)	No (2)	Mixed (1Y, 1N)

Abbreviations: BE = Belgium, DE = Denmark, ES = Estonia, FR = France, GE = Germany, IT = Italy, NL = the Netherlands, PO = Portugal, SL = Slovenia, SP = Spain, SW = Sweden, UK = the United Kingdom, Y = yes, N = no, UNK = unknown, TVU = transvaginal ultrasound, EMB = endometrial biopsy.

Endometrial surveillance with transvaginal ultrasound is recommended in all available guidelines, while indications for performing endometrial biopsy vary. Although ineffective, surveillance of the ovaries—mostly through transvaginal ultrasound, sometimes combined with the tumor marker CA125—is reported in most countries, though not universally implemented.

MDTs are recommended in the national guidelines of a few countries. In many others, either no recommendation exists or responses from gynecologists are mixed, reflecting a lack of standardization in clinical practice.

4. In clinical practice: surveillance strategies

In practice, the preferred setting for gynecological surveillance in women with Lynch syndrome varies widely across European countries (Table 2). Surveillance is most often conducted in university hospitals and specialized hereditary cancer centers. Although no international classification for specialized hereditary cancer centers exists, we defined them as a clinic demonstrating expertise through specialized outpatient care and/or active scientific research, for example via an endowed chair or professorship in this field. Some gynecologists reported surveillance being performed in general hospitals or by private practicing gynecologists.

**Table 2:** Overview of gynecological surveillance strategies in women with Lynch syndrome in clinical practice

	Clinical setting for gynecological surveillance	Surveillance starting age	Surveillance stopping age	Interval between surveillance moments (months)
BE (4)	SHCC (1), UH (2), GH (1)	First visit (3), 35 y/o (1)	No (4)	6 (1), 12 (3)
DE (3)	UH (1), GH (2)	35 y/o (3)	No (3)	24 (3)
ES (2)	UH (2)	First visit (2)	No (2)	6 (1), 12 (1)
FR (2)	SHCC (2)	35 y/o (2)	No (2)	12 (2)
GE (3)*	Private practicing gynecologist (2)	30 y/o (2)	No (2)	12 (2)
IT (4)	SHCC (1), UH (3)	First visit (1), 30 y/o (1), 35 y/o (1), 40 y/o (1)	No (4)	12 (4)
NL (7)	SHCC (2), UH (2), GH (2), other (1)	First visit (1), 40 y/o (6)	60 y/o (4), 70 y/o (1), No (2)	12 (7)
PO (1)	SHCC	35 y/o	70 y/o	6
SL (2)	UH (2)	First visit (2)	No (2)	12 (2)
SP (2)	UH (2)	35 y/o (2)	No (2)	12 (2)
SW (1)	SHCC	30 y/o	No	12
UK (2)	SHCC (1), UH (1)	First visit (1), 35 y/o (1)	No (2)	12 (2)

*= One respondent does not inform patients about the possibility of surveillance. Abbreviations: BE = Belgium, DE = Denmark, ES = Estonia, FR = France, GE = Germany, IT = Italy, NL = the Netherlands, PO = Portugal, SL = Slovenia, SP = Spain, SW = Sweden, UK = the United Kingdom, GH = general hospital, SHCC = specialized hereditary cancer center, UH = university hospital, y/o = years old.

The starting age for surveillance varies between 30 and 40 years or is initiated at the first gynecological Lynch-related outpatient visit. There is no uniform stopping age of gynecological surveillance. Most gynecologists use a 12-months surveillance interval, although shorter (6 months) and longer (24 months) intervals are used.

5. In clinical practice: management of cancer prevention

Nearly all gynecologists report discussing risk-reducing hysterectomy with women with Lynch syndrome to lower their risk of endometrial cancer (Table 3). Currently there is considerable variation between and within countries on the indication of a risk-reducing hysterectomy in case of colorectal surgery. The use of preventive progestin therapy to reduce endometrial cancer risk appears limited and heterogeneous. While some respondents consistently recommended preventive progestin use—either routinely or in the presence of specific risk factors such as obesity—others stated that it was not advised.

**Table 3:** Overview of gynecological cancer prevention strategies in women with Lynch syndrome in clinical practice

	Risk-reducing hysterectomy discussed	Consideration of risk-reducing hysterectomy in case of colorectal surgery	Preventive progestin therapy advised to reduce EC	Risk-reducing BSO discussed	HRT advised after BSO	Healthy BMI advised
BE (4)	Yes (4)	Yes (2), No (1), UNK (1)	No (3) Yes, if risk factors (1)	Yes (3), advised 45 y/o (2), 50 y/o (1) No (1)	No (1) Yes, if menopausal symptoms (3)	Always (2), most of the time (2)
DE (3)	Mixed (2Y, 1N)	Yes (2), UNK (1)	No (2) Yes, if risk factors (1)	Yes (2), advised 50 y/o (1) No (1)	Yes, 35 y/o (1) Yes, if menopausal symptoms (2)	Most of the time (2), rarely (1)
ES (2)	Yes (2)	Yes (1), No (1)	Yes, always (1) Yes, if risk factors (1)	Yes (2)	Yes, if menopausal symptoms (2)	Always (2)
FR (2)	Yes (2)	Yes (1), No (1)	No (1) Yes, always (1)	Yes (2), advised 40 (1) or 40-50 y/o gPV-depending (1)	Yes, 40 y/o (2)	Most of the time (2)
GE (3)	Yes (3)	Yes (1), UNK (2)	No (1) UNK (2)	Yes (3), advised 40 y/o (2)	Yes, 40 y/o (2) Yes, if menopausal symptoms (1)	Always (2), most of the time (1)
IT (4)	Yes (4)	Yes (3), No (1)	No (2) Yes, if risk factors (2)	Yes (4), advised 43 y/o (1), 45 y/o (1)	Yes, 40 y/o (1), 43 y/o (1) Yes, if menopausal symptoms (2)	Always (4)
NL (7)	Yes (7)	Yes (7)	No (5) Yes, always (1) Yes, if risk factors (1)	Yes (6), advised 45 y/o (1), 50 y/o (2) No (1)	Yes, 40 y/o (5), 45 y/o (1) Yes, if menopausal symptoms (1)	Always (3), most of the time (4)
PO (1)	Yes	Yes	No	Yes	Yes, 45 y/o	Always
SL (2)	Yes (2)	No (2)	No (2)	Yes, advised 50 y/o (2)	Yes, if menopausal symptoms (2)	Always (1), most of the time (1)
SP (2)	Yes (2)	Yes (2)	Yes, always (1) Yes, if risk factors (1)	Yes (2), advised 45 y/o (1)	Yes, 40 y/o (2)	Always (2)
SW (1)	Yes	Yes	Yes, always	Yes	Yes, 35 y/o	Most of the time
UK (2)	Yes (2)	Yes (2)	Yes, always (2)	Yes (2), advised 45 y/o (1)	Yes (2)	Always (2)

Abbreviations: EC = endometrial cancer, BSO = bilateral salpingo-oophorectomy, HRT = hormone replacement therapy, BMI = body mass index, BE = Belgium, DE = Denmark, ES = Estonia, FR = France, GE = Germany, IT = Italy, NL = the Netherlands, PO = Portugal, SL = Slovenia, SP = Spain, SW = Sweden, UK = the United Kingdom, Y = yes, N = no, UNK = unknown, y/o = years old, gPV = germline pathogenic variant.

Risk-reducing BSO is discussed in most countries, with the timing of the procedure typically ranging from 40 to 50 years of age. When performed, hormone replacement therapy (HRT) is generally advised, particularly for women experiencing menopausal symptoms. In some cases, gynecologists recommend age-based initiation of HRT, typically between 35 and 45 years following BSO.

6. In clinical practice: treatment of (pre)malignancies

The management of endometrial hyperplasia—both with and without atypia—varied across European countries (Table 4). Hysterectomy for hyperplasia without atypia was generally not performed, although some mixed opinions were reported in several countries.

**Table 4:** Overview of gynecological treatment strategies in women with Lynch syndrome in clinical practice

	Hysterectomy in case of endometrial hyperplasia without atypia	BSO in case of endometrial hyperplasia without atypia	Hysterectomy in case of endometrial atypia	BSO in case of hyperplasia with atypia	Preferred manner of surgery
BE (4)	Mixed (3N, 1UNK)		Yes (4)	Yes, age-depending (4) 45 y/o (2) 50 y/o (2), gPV-depending (1)	Laparoscopic (3) Robotic (1)
DE (3)	Mixed (1Y, 2N)	Yes, age-depending 40 y/o (1)	Yes (3)	Yes, always (2) Yes, age-depending 40 y/o (1)	Laparoscopic (1) Robotic (2)
ES (2)	Yes (2)	Yes, age-depending 45 y/o (2), gPV-depending (1)	Yes (2)	Yes, age-depending 45 y/o (2) gPV-depending (1)	Laparoscopic (2)
FR (2)	No (2)		Yes (2)	Yes, age-depending (2) 40 y/o (1), gPV-depending (1) 45 y/o (1)	Laparoscopic (2)
GE (3)	Mixed (1Y, 2N)	Yes, age-depending 57 y/o (1)	Mixed (1Y, 2N)	Yes, age-depending 57 y/o (1)	Laparoscopic (3)
IT (4)	Mixed (2Y, 1N, 1UNK)	Yes, always (1), gPV-depending (1)	Yes (4)	Yes, always (2) Yes, age-depending 40 y/o (2)	Laparoscopic (4)
NL (7)	Mixed (2Y, 5N)	Yes, age-depending (2) 40 y/o (1), gPV-depending (1) 45 y/o (1)	Yes (7)	Yes, age-depending (6) 40 y/o (1), gPV-depending (1) 45 y/o (3) 48 y/o (1) 50 y/o (1), gPV-depending (1) Yes, gPV-depending (1)	Laparoscopic (7)
PO (1)	No		Yes	Yes, age-depending 45 y/o, gPV-depending	Laparoscopic
SL (2)	Mixed (1Y, 1N)	Yes, age-depending 50 y/o (1)	Yes (2)	Yes, age-depending 50 y/o (2)	Laparoscopic (2)
SP (2)	Mixed (1Y, 1N)	Yes, age-depending 42 y/o (1)	Yes (2)	Yes, age-depending (2) 40 y/o (1) 45 y/o (1)	Laparoscopic (1) *atypia: robotic with sentinel node Vaginal (1)
SW (1)	No		Yes	Yes, age-depending 40 y/o, gPV-depending	Laparoscopic
UK (2)	No (2)		Yes (2)	Yes, gPV-depending (2) 45 y/o (1)	Laparoscopic or robotic (2)

Abbreviations: BSO = bilateral salpingo-oophorectomy, BE = Belgium, DE = Denmark, ES = Estonia, FR = France, GE = Germany, IT = Italy, NL = the Netherlands, PO = Portugal, SL = Slovenia, SP = Spain, SW = Sweden, UK = the United Kingdom, Y = yes, N = no, UNK = unknown, y/o = years old, gPV = germline pathogenic variant.

In patients with endometrial hyperplasia with atypia, nearly all respondents reported performing a hysterectomy. A BSO was also frequently recommended, typically in an age-dependent manner (ranging from 40 to 57 years) or based on the specific gPV.

The preferred surgical approach is minimally invasive, with laparoscopic surgery being the most commonly reported technique.

7. Patient participation

The presence of Lynch-related patient associations, as well as referral practices, differed between gynecologists (Supplementary File 2).

## Discussion

This cross-sectional survey provides an overview of the clinical gynecological management of women with Lynch syndrome across Europe. We observed substantial variation in clinical gynecological practice of women with Lynch syndrome among gynecologists, both across and within countries. The inconsistency in gynecological care provided in this high-risk population is undesirable for patients.

Our survey demonstrates that national guidelines on the gynecological management of women with Lynch syndrome are absent in a considerable number of European countries. In others, awareness and implementation of such guidelines remain variable and incomplete. This emphasizes the urgent need for standardized, uniform European guidelines to optimize gynecological care for women with Lynch syndrome. Comparable European ERN GENTURIS guidelines already exist for other hereditary cancer syndromes, such as Li-Fraumeni syndrome, *PTEN* Hamartoma Tumour Syndrome (PHTS), and constitutional mismatch repair deficiency (CMMRD) [20–22].

A possible explanation for the limited awareness of the gynecological guideline may be that existing Lynch syndrome guidelines have predominantly focused on the prevention of colorectal cancer and were primarily written and distributed among gastrointestinal-focused medical professionals [7, 8]. This may have resulted in limited awareness and implementation among gynecologists. Therefore, we recommend the development of European multidisciplinary guidelines—created by both gynecologists and gastrointestinal-focused health care professionals as well as Lynch syndrome patient associations—that also address the gynecological management of women with Lynch syndrome.

Patients with rare hereditary diseases often experience a lack of coordination in multidisciplinary care, as care is typically organized separately by various medical specialties [23]. Uncoordinated multidisciplinary care could have an impact on patients‘ physical and psychosocial well-being and could lead to diagnostic errors, delays in diagnosis or treatment, and insufficient or outdated information about the disease [24, 25]. Figure 1 illustrates the diversity in the type of clinic gynecological care for Lynch carriers across Europe is provided. When surveillance is offered, the majority (73%) of gynecologists agree that care should be provided in a specialized hereditary cancer center or university hospital. This could contribute to more consistent gynecological care in women with Lynch syndrome across Europe. However, all gynecologists contributing to our survey were working in an expert center, which may have biased this outcome. We do not have data on the proportion of patients seen in non-specialized clinics by non-specialized physicians. Care provided in these settings may differ even more from what is reported in this study.

Local MDTs are held in hospitals of twenty (61%) of the responding gynecologist, with half of the patients being discussed only when indicated by a medical specialist. MDTs have been shown to improve adherence to clinical guidelines and can have a significant influence on management plans, process outcomes, and patients’ outcomes [26–28]. To standardize and optimize care in women with Lynch syndrome, there should at least be an opportunity to discuss patients during a local MDT. When this is unfeasible within their own hospital, regional, national or international MDTs composed of medical specialist in a reference network could serve as an alternative for discussing (complex) cases [29, 30].

Gynecologists offer endometrial surveillance across a wide range of ages in clinical practice. Current guidelines and literature are inconsistent regarding the recommended starting and stopping ages for surveillance, including differences based on the affected MMR-gene [8, 9, 31]. In our opinion, all women should know to contact their gynecologists in the event of irregular of postmenopausal bleeding. Ovarian surveillance is not recommended due to its lack of effectiveness for early detection and mortality reduction, and therefore should not be performed [12, 13]. There is limited evidence to support gynecological surveillance in Lynch syndrome and, no mortality benefit has been demonstrated [10]. Therefore, prospective studies should be established and their findings should be incorporated in standardized European guidelines to help ensure uniform management of these issues across countries.

We observed large heterogeneity in clinical practice of preventive progestin therapy to reduce endometrial cancer risk. Oral contraceptives and levonorgestrel-releasing intrauterine system (LNG-IUS) reduce the risk of endometrial cancer and, to a lesser extent, ovarian cancer in the general population [32–34]. The relative risks of developing endometrial and ovarian cancer among LNG-IUS users were 0.22 (95% confidence interval (CI): 0.13-0.40), and 0.53 (95% CI: 0.32-0.88), respectively, in a large cohort of 105,000 women [35]. Use of hormonal contraceptives is associated with reduced endometrial cancer risk among women with Lynch syndrome [36, 37]. Therefore, progestin therapy could be considered in women wishing to preserve fertility or those not willing to undergo risk-reducing hysterectomy.

Maintaining a healthy body mass index is recommended by most gynecologists. Up to now, unlike in sporadic endometrial cancer, no significant association has been observed between weight status or weight change and the risk of endometrial cancer in women with Lynch syndrome [38]. In contrast, the risk of colorectal cancer is significantly higher among Lynch syndrome carriers living with obesity [38, 39]. Furthermore, obesity is associated with an increased risk of perioperative and postoperative complications, which reinforces the importance of lifestyle counselling in this high-risk population [40].

Recommendations on risk-reducing BSO vary significantly. Recent studies suggest a lower risk of ovarian cancer in women with Lynch syndrome than previously anticipated. The cumulative incidence of ovarian cancer by age 65 is 8.0%, 10.6%, and 2.9% for gPVs in *MLH1*, *MSH2*, and *MSH6*, respectively, with a median age of cancer onset at 49, 47, and 65 years and low mortality rates [3, 41]. Carriers of gPVs in *PMS2* do not appear to be at higher risk for ovarian cancer compared to the general population. This suggests that the affected MMR-gene should be considered a key factor in the decision to perform a BSO, rather than relying on the woman’s age.

Premenopausal women should be offered HRT after BSO to prevent premature surgical menopause, until at least natural menopause age (51 years) [9]. This prevents the occurrence of menopausal symptoms, psychosexual problems, cardiovascular disease, osteoporosis, and cognitive disorders [17]. The survey reveals significant heterogeneity of indications for HRT, with it typically being suggested only when menopausal symptoms occur. Attention to HRT in the general population is increasing, which may allow for clearer recommendations in the future.

The Manchester International Consensus Group recommends that risk-reducing colorectal or surgery for colorectal cancer and gynecological surgery is carried out at the same time when possible [9]. This prevents multiple surgeries. A hysterectomy could affect future colonoscopies. A meta-analysis of 5,947 women reported lower colonoscopy completion rate (87.1% vs 95.5%, odds ratio, 0.28; 95% CI: 0.16-0.49) in women with a history of a hysterectomy [42]. However, a 2024 study, in which 52.3% of hysterectomies were performed laparoscopically, reported a lower cecal intubation rate and a higher frequency of technically challenging colonoscopies, yet still achieved a high overall completion rate of 94.4% [43]. Additionally, opportunistic salpingectomy may be considered during abdominal surgery, given its association with reduced ovarian cancer risk in the general population [44]. Given these considerations, centralized care for women with Lynch syndrome, including access to MDTs, is crucial to support individualized decision-making and to optimize both cancer prevention and surveillance strategies.

This study provides a complete overview of gynecological clinical management in women with Lynch syndrome across Europe. The response rate of participants was 57%, which is relatively high for a survey. However, this overview has several limitations. First, 94% of the responding gynecologists worked in a university hospital, and 73% of them identified their clinic as a specialized hereditary cancer center. In addition, gynecologists working in such specialized centers are likely to have above-average interest and knowledge regarding Lynch syndrome. This may have influenced their responses compared with those gynecologists not working in a specialized hereditary cancer center. As illustrated in Table 2, gynecological care is provided across various clinical settings, with notable variations both within and between countries. The majority of surveyed gynecologists advocate for care to be delivered in specialized clinics or university hospitals, although some suggest that it could also be provided in general hospitals or by private practicing gynecologists. It may be inferred that gynecologists in non-specialized settings are potentially less familiar with guidelines and recommendations compared to our respondents, of whom the majority are based in university hospitals.

Second, the 33 participating gynecologists were employed at 25 unique hospitals, which may have introduced selection bias and affected the representativeness of the results. Third, we aimed to give a clear overview in the gynecological care in women with Lynch syndrome across Europe. ERN GENTURIS includes centers in 22 European Union member states, Norway, and supporting partners in the United Kingdom, of which gynecologists from twelve different countries replied.

This cross-sectional survey among gynecologists demonstrates substantial variation in the gynecological management of women with Lynch syndrome across Europe. Clinical care practices differ not only between countries but also within individual countries.

The establishment of multidisciplinary European guidelines, with input from gynecologists, is warranted to promote uniformity and facilitate consistent implementation across centers. We consider that that the establishment of MDTs in centers treating patients with Lynch syndrome will facilitate the effective implementation of these multidisciplinary guidelines and enhance personalized care. ERN GENTURIS has the expertise and network necessary to lead the development of such multidisciplinary Lynch syndrome guidelines.

## Supplementary Information

Below is the link to the electronic supplementary material.

Supplementary Material 1

Supplementary Material 2

## Data Availability

No datasets were generated or analysed during the current study.

## References

[1] Lynch HT, Lynch PM, Lanspa SJ, Snyder CL, Lynch JF, Boland CR (2009) Review of the Lynch syndrome: history, molecular genetics, screening, differential diagnosis, and medicolegal ramifications. Clin Genet 76(1):1–18 19659756 10.1111/j.1399-0004.2009.01230.x PMC2846640

[2] Biller LH, Creedon SA, Klehm K, Yurgelun MB (2022) Lynch Syndrome-Associated cancers beyond colorectal cancer. Gastrointest Endosc Clin N Am 32(1):75–93 34798988 10.1016/j.giec.2021.08.002

[3] Dominguez-Valentin M, Haupt S, Seppälä TT, Sampson JR, Sunde L, Bernstein I et al (2023) Mortality by age, gene and gender in carriers of pathogenic mismatch repair gene variants receiving surveillance for early cancer diagnosis and treatment: a report from the prospective Lynch syndrome database. EClinicalMedicine. 58:101909 37181409 10.1016/j.eclinm.2023.101909 PMC10166779

[4] Castillo-Iturra J, Sánchez A, Balaguer F (2024) Colonoscopic surveillance in Lynch syndrome: guidelines in perspective. Fam Cancer 23(4):459–68 39066849 10.1007/s10689-024-00414-y PMC11512898

[5] van Leerdam ME, Roos VH, van Hooft JE, Balaguer F, Dekker E, Kaminski MF et al (2019) Endoscopic management of Lynch syndrome and of familial risk of colorectal cancer: European Society of Gastrointestinal Endoscopy (ESGE) guideline. Endoscopy 51(11):1082–93 31597170 10.1055/a-1016-4977

[6] Monahan KJ, Bradshaw N, Dolwani S, Desouza B, Dunlop MG, East JE et al (2020) Guidelines for the management of hereditary colorectal cancer from the British Society of Gastroenterology (BSG)/Association of Coloproctology of Great Britain and Ireland (ACPGBI)/United Kingdom Cancer Genetics Group (UKCGG). Gut. 69(3):411–44 31780574 10.1136/gutjnl-2019-319915 PMC7034349

[7] Seppälä TT, Latchford A, Negoi I, Sampaio Soares A, Jimenez-Rodriguez R, Sánchez-Guillén L, et al. (2021) European guidelines from the EHTG and ESCP for Lynch syndrome: an updated third edition of the Mallorca guidelines based on gene and gender. Br J Surg 108(5):484-98. 10.1002/bjs.11902 PMC10364896 34043773

[8] Richtlijnendatabase. Erfelijke darmkanker: Lynch syndroom, polyposis en familiair darmkanker 2025 [cited 2025 29th of October]. Available from: https://richtlijnendatabase.nl/richtlijn/erfelijke_darmkanker_lynch_syndroom_polyposis_en_familiair_darmkanker/startpagina_richtlijn_erfelijke_darmkanker_lynch_syndroom_polyposis_en_familiair_darmkanker.html.

[9] Crosbie EJ, Ryan NAJ, Arends MJ, Bosse T, Burn J, Cornes JM et al (2019) The Manchester International Consensus Group recommendations for the management of gynecological cancers in Lynch syndrome. Genet Med 21(10):2390–400 30918358 10.1038/s41436-019-0489-y PMC6774998

[10] Lim N, Hickey M, Young GP, Macrae FA, Kelly C (2022) Screening and risk reducing surgery for endometrial or ovarian cancers in Lynch syndrome: a systematic review. Int J Gynecol Cancer 32(5):646–55 35437274 10.1136/ijgc-2021-003132 PMC9067008

[11] Pakish JB, Lu KH, Sun CC, Burzawa JK, Greisinger A, Smith FA et al (2016) Endometrial Cancer Associated Symptoms: A Case-Control Study. J Womens Health (Larchmt). 25(11):1187–92 27254529 10.1089/jwh.2015.5657 PMC5116765

[12] Buys SS, Partridge E, Black A, Johnson CC, Lamerato L, Isaacs C et al (2011) Effect of screening on ovarian cancer mortality: the Prostate, Lung, Colorectal and Ovarian (PLCO) Cancer Screening Randomized Controlled Trial. Jama. 305(22):2295–303 21642681 10.1001/jama.2011.766

[13] Oei AL, Massuger LF, Bulten J, Ligtenberg MJ, Hoogerbrugge N, de Hullu JA (2006) Surveillance of women at high risk for hereditary ovarian cancer is inefficient. Br J Cancer 94(6):814–9 16495917 10.1038/sj.bjc.6603015 PMC2361371

[14] Snowsill TM, Coelho H, Morrish NG, Briscoe S, Boddy K, Smith T et al (2024) Gynaecological cancer surveillance for women with Lynch syndrome: systematic review and cost-effectiveness evaluation. Health Technol Assess 28(41):1–228 39246007 10.3310/VBXX6307 PMC11403379

[15] Schmeler KM, Lynch HT, Chen LM, Munsell MF, Soliman PT, Clark MB et al (2006) Prophylactic surgery to reduce the risk of gynecologic cancers in the Lynch syndrome. N Engl J Med 354(3):261–9 16421367 10.1056/NEJMoa052627

[16] Madhvani K, Garcia SF, Fernandez-Felix BM, Zamora J, Carpenter T, Khan KS (2022) Predicting major complications in patients undergoing laparoscopic and open hysterectomy for benign indications. CMAJ 194(38):E1306-e17 36191941 10.1503/cmaj.220914 PMC9529570

[17] Secoșan C, Balint O, Pirtea L, Grigoraș D, Bălulescu L, Ilina R (2019) Surgically Induced Menopause-A Practical Review of Literature. Medicina (Kaunas). 55(8):482 31416275 10.3390/medicina55080482 PMC6722518

[18] Stuursma A, van Driel CMG, Wessels NJ, de Bock GH, Mourits MJE (2018) Severity and duration of menopausal symptoms after risk-reducing salpingo-oophorectomy. Maturitas 111:69–76 29673834 10.1016/j.maturitas.2018.01.012

[19] ERN-GENTURIS. About ERN GENTURIS [cited 2025 29th October]. Available from: https://www.genturis.eu/l=eng/about-us/about-ern-genturis.html.

[20] Frebourg T, Bajalica Lagercrantz S, Oliveira C, Magenheim R, Evans DG (2020) Guidelines for the Li-Fraumeni and heritable TP53-related cancer syndromes. Eur J Hum Genet 28(10):1379–86 32457520 10.1038/s41431-020-0638-4 PMC7609280

[21] Tischkowitz M, Colas C, Pouwels S, Hoogerbrugge N (2020) Cancer surveillance guideline for individuals with PTEN hamartoma tumour syndrome. Eur J Hum Genet 28(10):1387–93 32533092 10.1038/s41431-020-0651-7 PMC7608293

[22] Colas C, Guerrini-Rousseau L, Suerink M, Gallon R, Kratz CP, Ayuso É et al (2024) ERN GENTURIS guidelines on constitutional mismatch repair deficiency diagnosis, genetic counselling, surveillance, quality of life, and clinical management. Eur J Hum Genet. 32(12):1526–41 39420201 10.1038/s41431-024-01708-6 PMC11607302

[23] Walton H, Ng PL, Simpson A, Bloom L, Chitty LS, Fulop NJ et al (2023) Experiences of coordinated care for people in the UK affected by rare diseases: cross-sectional survey of patients, carers, and healthcare professionals. Orphanet J Rare Dis. 18(1):364 37996938 10.1186/s13023-023-02934-9 PMC10668407

[24] von der Lippe C, Diesen PS, Feragen KB (2017) Living with a rare disorder: a systematic review of the qualitative literature. Mol Genet Genomic Med 5(6):758–73 29178638 10.1002/mgg3.315 PMC5702559

[25] Simpson A, Bloom L, Fulop NJ, Hudson E, Leeson-Beevers K, Morris S et al (2021) How are patients with rare diseases and their carers in the UK impacted by the way care is coordinated? An exploratory qualitative interview study. Orphanet J Rare Dis 16(1):76 33568181 10.1186/s13023-020-01664-6 PMC7874609

[26] Kočo L, Weekenstroo HHA, Lambregts DMJ, Sedelaar JPM, Prokop M, Fütterer JJ et al (2021) The Effects of Multidisciplinary Team Meetings on Clinical Practice for Colorectal, Lung, Prostate and Breast Cancer: A Systematic Review. Cancers (Basel). 13(16):4159 34439312 10.3390/cancers13164159 PMC8394238

[27] Taplin SH, Weaver S, Salas E, Chollette V, Edwards HM, Bruinooge SS et al (2015) Reviewing cancer care team effectiveness. J Oncol Pract. 11(3):239–46 25873056 10.1200/JOP.2014.003350 PMC4438110

[28] Pillay B, Wootten AC, Crowe H, Corcoran N, Tran B, Bowden P et al (2016) The impact of multidisciplinary team meetings on patient assessment, management and outcomes in oncology settings: a systematic review of the literature. Cancer Treat Rev 42:56–72 26643552 10.1016/j.ctrv.2015.11.007

[29] Engels M, Urbanczyk K, Hölzenspies J, Röhl C, Geverink N, Hoogerbrugge N (2025) The European Reference Network on Genetic Tumour Risk Syndromes (ERN GENTURIS): benefits for patients, families, and health care providers. Fam Cancer 24(2):33 40159592 10.1007/s10689-025-00457-9 PMC11955429

[30] Varde A, McVeigh T, Cuthill V, Brady AF, DeSouza B, Latchford A et al (2025) Addressing uncertainty in hereditary colorectal cancer: the role of a regional expert multidisciplinary team meeting. Fam Cancer 24(1):26 40045045 10.1007/s10689-025-00451-1 PMC11882607

[31] Concin N, Matias-Guiu X, Vergote I, Cibula D, Mirza MR, Marnitz S et al (2021) ESGO/ESTRO/ESP guidelines for the management of patients with endometrial carcinoma. Int J Gynecol Cancer 31(1):12–39 33397713 10.1136/ijgc-2020-002230

[32] Iversen L, Sivasubramaniam S, Lee AJ, Fielding S, Hannaford PC (2017) Lifetime cancer risk and combined oral contraceptives: the Royal College of General Practitioners’ Oral Contraception Study. Am J Obstet Gynecol. 216(6):580 10.1016/j.ajog.2017.02.002 28188769

[33] Orbo A, Vereide A, Arnes M, Pettersen I, Straume B (2014) Levonorgestrel-impregnated intrauterine device as treatment for endometrial hyperplasia: a national multicentre randomised trial. BJOG 121(4):477–86 24286192 10.1111/1471-0528.12499 PMC4155866

[34] Kim MK, Seong SJ, Kim JW, Jeon S, Choi HS, Lee IH et al (2016) Management of Endometrial Hyperplasia With a Levonorgestrel-Releasing Intrauterine System: A Korean Gynecologic-Oncology Group Study. Int J Gynecol Cancer. 26(4):711–5 26905333 10.1097/IGC.0000000000000669

[35] Jareid M, Thalabard JC, Aarflot M, Bøvelstad HM, Lund E, Braaten T (2018) Levonorgestrel-releasing intrauterine system use is associated with a decreased risk of ovarian and endometrial cancer, without increased risk of breast cancer Results from the NOWAC Study. Gynecol Oncol. 149(1):127–32 29482839 10.1016/j.ygyno.2018.02.006

[36] Dashti SG, Chau R, Ouakrim DA, Buchanan DD, Clendenning M, Young JP et al (2015) Female hormonal factors and the risk of endometrial cancer in Lynch syndrome. JAMA 314(1):61–71 26151267 10.1001/jama.2015.6789 PMC4688894

[37] Lu KH, Loose DS, Yates MS, Nogueras-Gonzalez GM, Munsell MF, Chen LM et al (2013) Prospective multicenter randomized intermediate biomarker study of oral contraceptive versus Depo-Provera for prevention of endometrial cancer in women with Lynch syndrome. Cancer Prev Res (Phila) 6(8):774–81 23639481 10.1158/1940-6207.CAPR-13-0020 PMC3737517

[38] Coletta AM, Peterson SK, Gatus LA, Krause KJ, Schembre SM, Gilchrist SC et al (2019) Energy balance related lifestyle factors and risk of endometrial and colorectal cancer among individuals with Lynch syndrome: a systematic review. Fam Cancer 18(4):399–420 31236808 10.1007/s10689-019-00135-7 PMC6863045

[39] Power RF, Doherty DE, Parker I, Gallagher DJ, Lowery MA, Cadoo KA (2024) Modifiable risk factors and risk of colorectal and endometrial cancers in Lynch syndrome: a systematic review and meta-analysis. JCO Precis Oncol 8:e2300196 38207227 10.1200/PO.23.00196

[40] Inci MG, Richter R, Woopen H, Rasch J, Heise K, Anders L et al (2020) Role of predictive markers for severe postoperative complications in gynecological cancer surgery: a prospective study (RISC-Gyn Trial). Int J Gynecol Cancer 30(12):1975–82 33246921 10.1136/ijgc-2020-001879

[41] Møller P, Seppälä TT, Ahadova A, Crosbie EJ, Holinski-Feder E, Scott R et al (2023) Dominantly inherited micro-satellite instable cancer - the four Lynch syndromes - an EHTG, PLSD position statement. Hered Cancer Clin Pract. 21(1):19 37821984 10.1186/s13053-023-00263-3 PMC10568908

[42] Clancy C, Burke JP, Chang KH, Coffey JC (2014) The effect of hysterectomy on colonoscopy completion: a systematic review and meta-analysis. Dis Colon Rectum 57(11):1317–23 25285700 10.1097/DCR.0000000000000223

[43] Hyldebrandt HK, Grindedal EM, Huppertz-Hauss G, Vitelli V, Johansen N, Stormorken AT (2024) The impact of hysterectomy on subsequent colonoscopy in women with Lynch Syndrome. Scand J Gastroenterol 59(8):1015–20 38946231 10.1080/00365521.2024.2366969

[44] Kahn RM, Gordhandas S, Godwin K, Stone RL, Worley MJ Jr, Lu KH et al (2023) Salpingectomy for the Primary Prevention of Ovarian Cancer: A Systematic Review. JAMA Surg. 158(11):1204–11 37672283 10.1001/jamasurg.2023.4164 PMC11185162

