# Correction: Hereditary gynecological cancer management in women with Lynch syndrome: a survey across Europe

**DOI:** 10.1007/s10689-026-00553-4

**Published:** 2026-04-22

**Authors:** Kevin J. J. Kwinten, Joanne A. de Hullu, ERN GENTURIS Survey Group, Helen Bolton, Monique M. A. Brood-van Zanten, Valentina Chiappa, Stefan Cosyns, Sergi Fernandez-Gonzalez, Angelique Flöter Rådestad, Sophie Frank, Rieke Gellner, Susanne Housmans, Nina Kovacevic, Iveta Mikeltadze, Sebastjan Merlo, Charlotte Møller, Johanna M. A. Pijnenborg, Kresten R. Petersen, Anna Myriam Perrone, Neil A. J. Ryan, Claire Saule, Rawand Salihi, Montserrat Serra Landete, Helena C van Doorn, Errico Zupi, Lena Steinkasserer, Nicoline Hoogerbrugge, Anne M. van Altena

**Affiliations:** 1 Department of Obstetrics & Gynecology, Radboud University Medical Center, Nijmegen, The Netherlands; 2 Department of Human Genetics, Radboud University Medical Center, Nijmegen, The Netherlands; 3 Department of Gynaecological Oncology, Cambridge University Hospitals NHS Foundation Trust, Cambridge, UK; 4 Department of Gynecology, The Netherlands Cancer Institute, Amsterdam, The Netherlands; 5 Ginecologia Chirurgica, IRCCS Istituto Nazionale Tumori, Milan, Italy; 6 Department of Gynaecologic Oncology, University Hospital UZ Brussel, Brussels, Belgium; 7 Gynaecological Department, Bellvitge University Hospital (IDIBELL), Universitat de Barcelona (UB), Barcelona, Spain; 8 Department of Women’s and Children’s Health, Karolinska Institutet, Stockholm, Sweden; 9 Department of Oncology and Genetics, Institut Curie, Paris, France; 10 Department of Gynecology and Obstetrics, Medical School Hannover, Hannover, Germany; 11 Department Obstetrics & Gynaecology, University Hospitals KU Leuven, Leuven, Belgium; 12 Department of Gynecological Oncology, Institute of Oncology, Ljubljana, Slovenia; 13 Department of Surgical and Gynaecological Oncology, Tartu University Hospital, Surgery Clinic, Tartu, Estonia; 14 Department of Gynecological Oncology, Institute of Oncology, Ljubljana, Slovenia; 15 Department of Obstetrics and Gynecology, Aarhus University Hospital, Aarhus, Denmark; 16 Department of Obstetrics & Gynecology, Radboud University Medical Center, Nijmegen, The Netherlands; 17 Department of Obstetrics and Gynecology, Herlev University Hospital, University of Copenhagen, Herlev, Denmark; 18 Division of Oncologic Gynecology, IRCCS Azienda Ospedaliero-Universitaria di Bologna, Bologna, Italy; 19 College of Medicine and Veterinary Medicine, The University of Edinburgh, Edinburgh, UK; 20 Department of Medical Oncology, Institut Curie, Paris, France; 21 Department of Obstetrics and Gynecology, Ghent University Hospital, Ghent, Belgium; 22 Obstetrics and Gynecology, Hospital Universitari Germans Trias i Pujol, Universitat Autònoma de Barcelona, Badalona, Spain; 23 Department of Gynecologic Oncology, Erasmus MC Cancer Institute, University Medical Centre Rotterdam, Rotterdam, The Netherlands; 24 Department of Molecular and Developmental Medicine, Obstetrics and Gynecological Clinic, University of Siena, 53100 Siena, Italy; 25 Department of Gynecology and Obstetrics, Hannover Medical School, Hannover, Germany

**Correction to: Familial Cancer** 10.1007/s10689-026-00546-3

In this article, the three authors' names were missing from the ERN GENTURIS Survey Group list. The authors' names and affiliations are given below:

1. Helena C van Doorn—Department of Gynecologic Oncology, Erasmus MC Cancer Institute, University Medical Centre Rotterdam, Rotterdam, The Netherlands
2. Errico Zupi—Department of Molecular and Developmental Medicine, Obstetrics and Gynecological Clinic, University of Siena, 53100 Siena, Italy
3. Lena Steinkasserer—Department of Gynecology and Obstetrics, Hannover Medical School, Hannover, Germany

The original article has been corrected.

