# Supplementary material for: Hereditary gynecological cancer management in women with Lynch syndrome: a survey across Europe
Source: Fam Cancer. 2026 Mar 31;25(2):36. doi: 10.1007/s10689-026-00546-3 (PMC13038461; doi:10.1007/s10689-026-00546-3)
Supplement: Supplementary file 1 — Supplementary Material 1 [file 10689_2026_546_MOESM1_ESM.docx]

**Supplementary File 1**

Dear participant,

We are grateful for your time in completing this survey. With your contribution, we aim to provide a clear and comprehensive overview of how care for women with Lynch syndrome is organized in Europe. If you have any questions or comments, please do not hesitate to contact the main researcher, Kevin Kwinten, at [kevin.kwinten@radboudumc.nl](mailto:kevin.kwinten@radboudumc.nl).

# General information

1. What is your gender?

Female/Male/I don’t want to say

1. What is your age? …
2. Have you obtained a PhD degree?

Yes/Yes, in the field of gynecologic oncology or hereditary cancer/No or not yet

1. What is your function?

General gynecologist/Gynecologist with interest in oncology/Gynecologic oncologist (completed fellowship)

1. How many years of experience do you have as a gynecologist? …
2. In what type of clinic do you work?

Private clinic/General hospital/University hospital

1. Are you (partly) working in a specialized hereditary cancer clinic? *We describe a specialized hereditary cancer clinic as a clinic where demonstrable expertise exists in the form of specialized outpatient care and/or active scientific research, for example, through an endowed chair or professorship in this field of expertise.*

Yes/No

1. In which country do you work?

Austria/Belgium/Cyprus/Czech Republic/Denmark/Estonia/Finland/France/Germany/Greece/ Hungary/Italy/Lativa/Lithuania/Luxembourg/Malta/Netherlands/Norway/Poland/Portugal/Slovenia/Spain/Sweden/United Kingdom

1. Is your clinic collaborating in research projects regarding Lynch syndrome?

Yes/No/I don’t know

1. How many new outpatient women with Lynch syndrome visit your clinic each year?

0-19/20 or more/I don’t know

1. Remarks of this topic: …

# Organization of care

1. Is care for women with Lynch syndrome in your country organized in certain centers (ie. university hospitals or familial cancer clinics)?

Yes/No/I don’t know

If yes, please specify

1. Is colorectal surveillance provided in the same center as where the patient has her gynecological care?

Yes, always/Yes, preferably/No/I don’t know

1. Are multidisciplinary meetings where Lynch patients can be discussed organized in your hospital?

Yes/No/I don’t know

If yes: Is every new Lynch patient discussed at this multidisciplinary meeting?

Yes, automatically/Yes, only if there are problems or questions to discuss/No/I don't know

If yes: Which medical disciplines are involved in these meetings?

Gynecology/Clinical genetics/Gastroenterology/Medical oncology/Pathology/Other/I don't know

If yes: How many multidisciplinary meetings do you have to discuss cases?

1 every 3-4 months/1 every 1-2 months/1 or more every month/Only when there is an indication for discussion/I don't know

1. To your opinion, who should be responsible for the gynecological care of women with Lynch syndrome?

General gynecologist/Gynecologist with interest in oncology/Gynecologic oncologist (= completed fellowship)/Other/I don't know

1. Remarks of this topic: …

# Guidelines

The following questions are related to the guidelines of your country. Questions about your clinical practice will follow.

1. Do you currently have a national guideline or protocol on care for Lynch syndrome?

Yes/No/I don’t know

If yes: When was the last update?

If yes: The following questions will follow.

1. Does the guideline contain recommendations on gynecological care?

Yes/No/I don’t know

If yes: Does the guideline mention gynecological surveillance in women with Lynch?

Yes/No/I don’t know

If yes: Does the guideline advise surveillance of the uterus?

Yes/No/I don’t know

If yes: How?

Ultrasound/Endometrial biopsy or microcurettage (always)/Endometrial biopsy or microcurettage (only if aberrancies during examination)/Hysteroscopy/Other

If yes: Does the guideline advise surveillance of the ovaries?

If yes: How?

Ultrasound/CA125/Other

If yes: Does the guideline mention gynecological risk reducing surgery in women with Lynch?

Yes/No/I don’t know

If yes: Does it advise risk reducing surgery?

Yes/No/I don’t know

If yes: Does the guideline recommend to discuss patients in multidisciplinary meetings?

Yes/No/I don’t know

If yes: Does the guideline mention which medical disciplines must be involved in these meetings?

Yes/No/I don’t know

If yes: Which medical disciplines must be involved?

Gynecology/Clinical genetics/Gastroenterology/Medical oncology/Pathology/Other/I don't know

If yes: Does the guideline contain different recommendations for gynecological care depending on the mutation the woman carries (ie. MHS6)?

Yes/No/I don’t know

1. If you have a national guideline, please provide us with the URL of your guideline. …
2. Remarks of this topic: …

# In clinical practice: surveillance strategies

The following questions will concern the care you as a physician provide for your patients with Lynch syndrome.

1. Do you inform patients that gynecological surveillance is an option for women with Lynch syndrome?

Yes/No

If yes: Where does gynecological surveillance preferably take place?

Specialized (hereditary) cancer center/University hospital/General hospital/Private clinic/Other/I don't know

If yes: Do you recommend to start gynecological surveillance at a specific age?

Yes/No. I recommend to start at the first time I speak about surveillance

If yes: At what specific age? …

If yes: Do you recommend to stop gynecological surveillance at a specific age?

Yes/No

If yes: At what specific age? …

If yes: What is the interval between surveillance visits? …

If yes: What types of diagnostics during surveillance do you primarily use?

Ultrasound/Endometrial biopsy or microcurettage (always)/Endometrial biopsy or microcurettage (only if aberrancies during examination)/Hysteroscopy/CA125

If yes: Is gynecological surveillance primarily performed in the same session as a coloscopy?

Yes, always/Yes, preferably/No/I don't know

Is gynecological examination primarily performed under sedation?

Yes/Yes, because it is performed during colonoscopy/No/I don't know

1. Remarks of this topic: …

# In clinical practice: management of cancer prevention

The following questions will concern the care you as a physician provide for your patients with Lynch syndrome.

1. Do you discuss prophylactic surgery of the uterus?

Yes/No/I don’t know

1. Do you discuss prophylactic surgery of the ovaries?

Yes, always/Yes, but I recommend it from certain age/No/I don't know

If yes, but I recommend it from certain age: At what age? …

1. Do you discuss prophylactic surgery of the fallopian tubes?

Yes/No/I don’t know

1. If you conduct prophylactic surgery, what is the preferred manner of surgery?

Laparoscopic/Robotic/Abdominal or open/Vaginal/I don't know

1. Do you explain that the salpingectomy is probably opportunistic because Lynch related ovarian cancers are mostly non-high grade serous carcinomas?

Yes/No/I don’t know

1. Do you advise preventive progestin therapy to reduce risk of endometrial cancer?

Yes, always/Yes, if risk factors (ie. obesity)/No/I don't know

If yes: What type of progestin?

Preferably progestin IUD/Preferably oral progestin/No preference

1. Do you advise hormone replacement therapy after removal of the ovaries?

Yes, age-related/Yes, only in case of menopausal complaints/No/I don't know

If yes, age-related: Starting from what age? …

If yes, age-related: Do you have a upper limit for the prescription of hormone replacement therapy?

Yes/No/I don’t know

If yes: What is the upper limit? …

1. Do you discuss sports as part of lifestyle to decrease risk of endometrial cancer?

Always/Most of the time/Rarely/Never/I don't know

1. Do you discuss weight reduction in case of high BMI to decrease risk of endometrial cancer?

Always/Most of the time/Rarely/Never/I don't know

1. Do you discuss healthy food as part of lifestyle to decrease risk of endometrial cancer?

Always/Most of the time/Rarely/Never/I don't know

1. Do you discuss the role of oral contraceptives to decrease risk of ovarian cancer?

Always/Most of the time/Rarely/Never/I don't know

1. Do you consider prophylactic hysterectomy in case of colorectal surgery?

Yes/No/I don’t know

1. Do you consider prophylactic oophorectomy in case of colorectal surgery?

Yes/No/I don’t know

1. In case of gynecological surgery, what is your minimum of interval between gynecological surgery and most recent colonoscopy?

0-6 months/7-12 months/More than 12 months/Not indicated/I don't know

1. Remarks of this topic: …

# In clinical practice: treatment of (pre)malignancies

The following questions will concern the care you as a physician provide for your patients with Lynch syndrome.

1. Do you advise hysterectomy in case of hyperplasia without atypia?

Yes/No/I don’t know

If yes: With or without bilateral salpingo-oophorectomy (BSO)?

With BSO/With BSO, age-depending/With BSO, mutation-depending/No BSO/I don't know

If with BSO, age-depending: At what age? …

1. Do you advise hysterectomy in case of hyperplasia with atypia?

Yes/No/I don’t know

If yes: With or without bilateral salpingo-oophorectomy (BSO)?

With BSO/With BSO, age-depending/With BSO, mutation-depending/No BSO/I don't know

If with BSO, age-depending: At what age? …

1. If you conduct therapeutic surgery, what is the preferred manner of surgery?

Laparoscopic/Robotic/Abdominal or open/Vaginal/I don't know

1. Remarks of this topic: …

# Patient participation

1. Do you have a Lynch syndrome patient association in your country?

Yes/No/I don’t know

If yes: Do you actively inform patients of the existence of this association?

Always/Most of the time/Rarely/Never/I don't know

1. Do clinicians actively participate in patient associations (ie. giving lectures)?

Yes/No/I don’t know

1. Remarks of this topic: …

# Contact details

1. If necessary, I may be contacted for an interview to further explain my answers.

Yes/No

1. I would like that my participation in this survey is mentioned in a publication in the future.

Yes/No

1. If any of above yes, please leave your e-mail address. …

We would like to thank you for participating in this survey. If you have any questions or comments, please do not hesitate to contact the main researcher, Kevin Kwinten, at [kevin.kwinten@radboudumc.nl](mailto:kevin.kwinten@radboudumc.nl).
