# Supplementary material for: Hereditary gynecological cancer management in women with Lynch syndrome: a survey across Europe
Source: Fam Cancer. 2026 Mar 31;25(2):36. doi: 10.1007/s10689-026-00546-3 (PMC13038461; doi:10.1007/s10689-026-00546-3)
Supplement: Supplementary file 2 — Supplementary Material 2 [file 10689_2026_546_MOESM2_ESM.docx]

**Supplementary File 2**

|  | Existence of national Lynch syndrome patient association | Referral to Lynch syndrome patient association by clinician |
| --- | --- | --- |
| **BE (4)** | Mixed (2Y, 2UNK) | Most of the time (1), rarely (1) |
| **DE (3)** | UKN (3) |  |
| **ES (2)** | No (2) |  |
| **FR (2)** | Yes (2) | Most of the time (1), rarely (1) |
| **GE (3)** | Mixed (2Y, 1UNK) | Always (2) |
| **IT (4)** | Mixed (2Y, 1N, 1UNK) | Most of the time (1), rarely (1) |
| **NL (7)** | Mixed (5Y, 2UNK) | Always (1), most of the time (1), rarely (3) |
| **PO (1)** | UNK |  |
| **SL (2)** | Yes (2) | Most of the time (1), rarely (1) |
| **SP (2)** | UNK (2) |  |
| **SW (1)** | UNK |  |

Supplementary table 1. Overview of patient participation opportunities for women with Lynch syndrome across countries. Abbreviations: BE = Belgium, DE = Denmark, ES = Estonia, FR = France, GE = Germany, IT = Italy, NL = the Netherlands, PO = Portugal, SL = Slovenia, SP = Spain, SW = Sweden, UK = the United Kingdom, Y = yes, N = no, UNK = unknown.
